# Voices in Images: Unveiling the Lived Realities of Adolescents with Disabilities in Ghana

**DOI:** 10.3390/ijerph23050678

**Published:** 2026-05-20

**Authors:** Josephine M. Kyei, Charles Ampong Adjei, Mary A. Asirifi, William Menkah, Prisca Ama Anima, Hellen Gateri, Reyna Parikh, Elizabeth Burgess-Pinto, Florence Naab

**Affiliations:** 1 School of Nursing and Midwifery, University of Ghana, Accra P.O. Box LG 43, Ghana; jmkyei@ug.edu.gh (J.M.K.);; 2 Faculty of Nursing, MacEwan University, Edmonton, AB T5J 4S2, Canada; 3 Department of Geography and Sustainability Science, University of Energy and Natural Resources Ghana, Sunyani P.O. Box 214, Ghana; 4 School of Social Work, Faculty of Health and Community Studies, MacEwan University, Edmonton, AB T5J 4S2, Canada

**Keywords:** disabilities, adolescents, Ghana, photovoice

## Abstract

**Highlights:**

**Public health relevance—How does this work relate to a public health issue?**

- Adolescents with disabilities constitute a substantial proportion of the 8% disability burden in Ghana.
- Yet, the perspectives of this marginalised population are often overlooked, influencing the design of context-specific interventions tailored to their specific needs.

**Public health significance—Why is this work of significance to public health?**

- This study highlights a critical evidence gap by exploring the socio-cultural challenges faced by adolescents with disabilities through a participatory photovoice approach.
- Based on these findings, programmes and policies targeting this marginalised population can be developed while meaningfully incorporating their voices.

**Public health implications—What are the key implications or messages for practitioners, policy makers and/or researchers in public health?**

- The findings show the need for culturally appropriate interventions that require the inclusion of the views of adolescents with disabilities.
- It also provides a basis for future research that may document the magnitude of the challenges faced by adolescents with disabilities in Ghana.

**Abstract:**

Ghana has a substantial disability burden with approximately 8% of the population, including adolescents, living with one form of disability or another. Despite this, the everyday experiences and challenges of adolescents with disabilities remain insufficiently documented. This study employed a phenomenological qualitative approach using photovoice methodology to explore the inner lives and often unvoiced experiences of adolescents with disabilities within their socio-cultural contexts. A total of fifty-four (54) adolescents aged 10–19 years with hearing, visual, and physical disabilities participated in the study. Participants were purposively selected to ensure maximum variation by sex, age and locality. The data were analysed manually using the photovoice data analyses procedure as proposed by Tsang. Three overarching themes emerged from the data: adversity, resilience, and social support. Participants used a range of visual images to represent their challenges, including images symbolising darkness, a stick lying on a bare floor, a coconut tree, heaps of sand, and stacks of wood logs. Images of chapel and group gatherings were also used to illustrate coping strategies and social support respectively. These findings underscore the need for the development of age-appropriate, resilience-focused interventions tailored to adolescents with disabilities in Ghana. it also highlights the need for larger community support networks and empowerment groups that meet the needs of adolescents with disabilities in Ghana.

## 1. Introduction

Adolescence refers to the transitional stage between childhood and adulthood, typically spanning ages 10 to 19 [1]. During this period, both boys and girls experience accelerated physical growth, significant biological changes, and increased awareness of their appearance alongside cognitive development [2]. Because of these rapid transformations, adolescence is considered a crucial life stage marked by major developmental tasks and notable challenges [3,4]. Numerous studies highlight that adolescents often struggle with academic demands, substance use, mental health issues, depression, suicidal ideation, and, in some cases, an elevated risk of violence or self-harm [5,6]. Furthermore, this period involves the formation of self-identity, which is influenced by personal exploration as well as social feedback from peers, family, and the broader community [7].

However, adolescents with disabilities tend to experience these difficulties more intensely, as they must cope with typical developmental challenges while also managing disability-related barriers. For instance, young people with disabilities are frequently marginalised and excluded within their communities [8]. In Ghana, adolescents with disabilities often face stigma, expressed through derogatory labelling, ableist attitudes, and social exclusion [8]. Similar findings have also been reported in Ethiopia [9].

Ghana faces a considerable disability burden [10]. Of its 30.8 million citizens, approximately 8%, including adolescents, live with various forms of disability [10], reflecting a notable increase from earlier estimates that placed national disability prevalence at 3% [11]. Despite the size of this population, the lived experiences of people with disabilities, particularly adolescents, remain insufficiently examined. Existing research in Ghana has largely concentrated on disability service delivery and the perspectives of caregivers [12,13]. In response to this gap, the present study adopted a photovoice approach to empower adolescents with disabilities to express their realities, challenges, and strengths through photography, thereby generating richer, participant-driven insights to inform policy, practice, and inclusive interventions. The aim of this study was to explore the lived realities and socio-cultural experiences of adolescents with disabilities in Ghana using photovoice methodology. The research questions guiding the study were: (1) How do adolescents with disabilities in Ghana perceive and describe their daily lived experiences? (2) What sources of resilience and social support do they identify?

## 2. Materials and Methods

### 2.1. Study Design

The study employed a phenomenological qualitative approach [14]. The appropriateness of this design stemmed from the constructivism paradigm, which enabled careful description, interpretation and analysis of participants’ inner life experiences instead of reflecting on their unspoken everyday experiences. It provides broader insights into the various events taking place in their lives.

### 2.2. Study Setting

This study was carried out across three regions of Ghana, namely Upper West, Ashanti, and Central which broadly reflect the northern, middle, and southern zones of the country. The regions were purposively selected because they have special schools that provide basic education for adolescents with diverse disabilities and were also accessible to the research team. Data were collected from four institutions: Wa Methodist School for the Blind, Jachie Training Centre for the Disabled, Ashanti School for the Deaf, and Cape Coast School for the Blind and Deaf. All these schools are funded by the Government of Ghana. This study is part of a broader research project [8].

### 2.3. Participants and Recruitment

Participants were eligible for inclusion if they: (1) were adolescents aged 10–19 years with hearing, visual, or physical disabilities; (2) were enrolled in any of the selected special schools within the study settings; and (3) provided consent to participate in the study. This ensured extensive inclusion of participants with different types of disabilities and diverse experiences. The age range was based on the operationalisation of adolescent definition by the World Health Organization [1]. However, adolescents with intellectual disabilities were excluded because we did not design or validate the photovoice methodology for this population, and none of the special schools involved in the data collection enrolled such students.

Data collection was conducted between July and October 2024. Recruitment of participants was undertaken following formal permission from the headteachers of the respective schools. The purpose of the study and confidentiality arrangements were clearly explained to prospective participants, and those who provided consent were subsequently interviewed. Assent was obtained from participants below 18 years of age, in addition to consent from their caregivers or teachers. For participants with hearing impairments, trained sign language interpreters who had signed confidentiality agreements with the research team facilitated the interpretation of both the interview guide and participants’ responses. The interviews were conducted in outdoor settings within the school premises, typically under trees.

### 2.4. Sampling and Sample Size Determination

Participants were purposively selected from three special schools in Ghana which ensured maximum variation by sex, age, district and locality. Overall, 54 participants took part in this study. Of the fifty-four, eighteen each were recruited from special schools in the northern, southern, and middle zones in Ghana. All participants agreed to take part in the study. The approach adopted ensured data saturation (i.e., when new interview provided no new information), which improved the quality of the findings [15]. The study employed four focus groups comprising eight members for the collective photovoice. A group of seven (7) to ten (10) photovoice participants has been suggested as an ideal size [16]. Participants were stratified by age to promote shared characteristics within groups and encourage open discussion. Specifically, participants aged 10–12 years were placed in one group, those aged 13–15 years in another, and participants aged 16–19 years in a separate group. Apart from the collective or group photovoice, individual photovoices were also collected to complement the group photovoices. The purpose for using both group and individual photovoice was to deepen and cross-check participants’ understanding of their lived experiences. Every respondent was given an opportunity to create his or her own photovoice. This enabled participants to share their disability experiences from different views. In all, four group and twenty-two individual photovoices were collected.

### 2.5. Data Collection Instrument

Photovoice methodology was employed for this study. This facilitated the phenomenological qualitative study design by allowing participants to capture their daily realities through photographs. Combining photovoice with phenomenology also creates rich layered data such as visual, narrative and reflective interpretations [17]. Cameras were provided to a group of adolescents with disabilities who were asked to take images that cognitively reflect their experiences [18]. The photographs were then used as catalysts for discussions. With photovoice, different stories are told, different photographs are captured, and different outcomes are sought. The heart of photovoice is the intermingling of images and words [19]. Evidence on core belief suggests that people experiencing a phenomenon understand the strengths and limitations of their situation better than outside experts [20].

### 2.6. Data Collection Procedure

The researchers explained to the participants using appropriate communication aids and assistive devices and assistants that they were to take photographs of objects, places, or situations that represented their day-to-day lived experiences as an adolescent living with disabilities. Given that participants who were blind were unable to capture images independently, caregivers/research assistants supported them by taking photographs based on the participants’ descriptions of the object. This was done in two folds; the group or collective photos were taken as well as individual photos. Both focus group and individual participants’ photovoices were collected to provide in-depth personal narratives/photographs with broader group insights to strengthen the research ability to influence social change. Furthermore, photovoice is designed to empower individuals and communities to identify and articulate what is important to them; thus, both group and individual photovoice processes help to reveal what truly matters [21]. To ensure accuracy and authenticity, the photographs accompanying the participants’ narratives were reviewed with the adolescents, allowing them to verify and validate their responses. In all, four groups and twenty-two individual photographs were produced.

### 2.7. Data Analysis

The data were analysed manually by authors CAA, WM and PAA following the procedure proposed by Tsang [22] for photovoice. The photovoice materials selected for analysis were justified on the basis of representativeness, data saturation, and depth of information. We took into account perspectives of both participants’ and researchers’ interpretations as well as recognising participants’ photographs and narratives as meaning of data during analysis [22]. The reason is not to distort participants’ interpretations but to generate theoretical explanations of the phenomenon under study. The analysis consisted of four main strategies including (a) photograph analysis based on researcher interpretation, (b) photograph interpretation based on participants’ interpretations, and (c) a cross-comparison (d) theorization.

First, an initial analysis of the photographs was conducted to generate preliminary insights into the phenomenon. This involved categorising the images, followed by iterative examination, re-categorization, and further review until data saturation was achieved. In the second stage, alternative interpretations of the photographs were developed based on participants’ explanations. As in the initial phase, both the photographs and accompanying narratives were systematically categorised and re-examined to derive themes, with the process continuing until saturation was reached (i.e., no new information emerged) [16]. The themes were derived using Hussey’s PHOTO acronym [23], which includes: What is described in the picture? What is happening in the picture? What the picture tells about participants’ lives? How can the picture improve participants’ lives? What does the analysis seek to understand about the world of disability from both the perspectives of participants and researchers? Following data saturation, the two sets of themes were compared. This involved systematic comparison of photographs with photographs, narratives with narratives, and cross-comparisons between photographs and narratives. Re-categorisation was also undertaken to generate additional themes. The analysis comprised multiple stages, including detailed examination of each photograph, synthesis of photographic findings, and integration of themes derived from both researchers and participants. Subsequently, the interpretations were used to theorise the emergent themes from the study. Notably, there were no clear differences between participants’ and researchers’ interpretations, which facilitated a smooth process of integration and theorisation. All images collected were analysed, and the entire analytical process was thoroughly documented.

### 2.8. Ethical Considerations

Ethical approval was provided by the University of Ghana medical Centre institutional Review board (Protocol No. UGMC/IRBREVIEW/020/24) and MacEwan University Research Ethics Board (File No: 102243) and the study was conducted according to the Declaration of Helsinki. We obtained assent from participants below the age of 18 years and written consent from their caregivers/teachers in the schools. Prior to the study, participants were informed that in situations where self-harm thoughts occur during the interview, the interview will be paused or stopped completely, and participants feelings will be acknowledged in a non-judgemental way, and the researchers will stay with participant until the necessary support/counselling is arranged for participant as required. Although a counsellor (the lead author) was available on site, her services were not utilised, as none of the participants experienced any psychological or emotional distress during data collection that necessitated professional intervention. Additionally, consent to include pictures in the publication was sought from participants and their caregivers/teachers/head teachers in the schools prior to the study. All data were anonymized to ensure participants’ confidentiality.

### 2.9. Trustworthiness

To ensure trustworthiness of the study, four criteria were adhered to; credibility, dependability, confirmability and transferability. Credibility was ensured by adopting various strategies, including prolonged engagement of participants and member checking [24]. Also, the preliminary findings were shared with two representatives of the participants to validate if they were consistent with their own experiences. Dependability and confirmability were ensured by detailed description of the research process and keeping field notes as well as photographs and voice records. Reflexivity was ensured by the researchers’ consciousness of their own biases and personal beliefs about the phenomenon [24].

## 3. Results

### 3.1. Sociodemographic Characteristic of the Participants

In total, 54 participants took part in the study, comprising 34 females and 20 males. Of these, 23 had visual impairments, 22 had hearing impairments, and 9 had physical disabilities. Nearly all participants reported that their disabilities were congenital, with the exception of four with visual impairments who indicated that their condition was acquired later in life.

Table 1 presents the researcher’s systematic thematic analysis of eight photovoice images submitted by adolescents with disabilities. The table is organised into three columns; Theme, Sample Photograph, and Researcher Interpretation and is structured around three overarching thematic categories that emerged from the analytical process: Challenging, Resilience, and Support Systems. Each theme presents a set of photographic Figures, which the researcher subjected to interpretive analysis grounded in the lived experiences of the study participants.

Table 2 presents the participants’ own interpretations of the same eight photovoice images, offering an emic perspective that highlights the subjective meanings attributed to the photographs by the adolescents themselves. The table is similarly structured around three columns, theme, sample photograph, and participants’ interpretations and is organised under five thematic categories: rejection, burden, challenging, hopefulness, and support system.

### 3.2. Theorisation of the Themes

#### 3.2.1. Adolescents with Disabilities as an Adversary Experience

**Figure 1:**
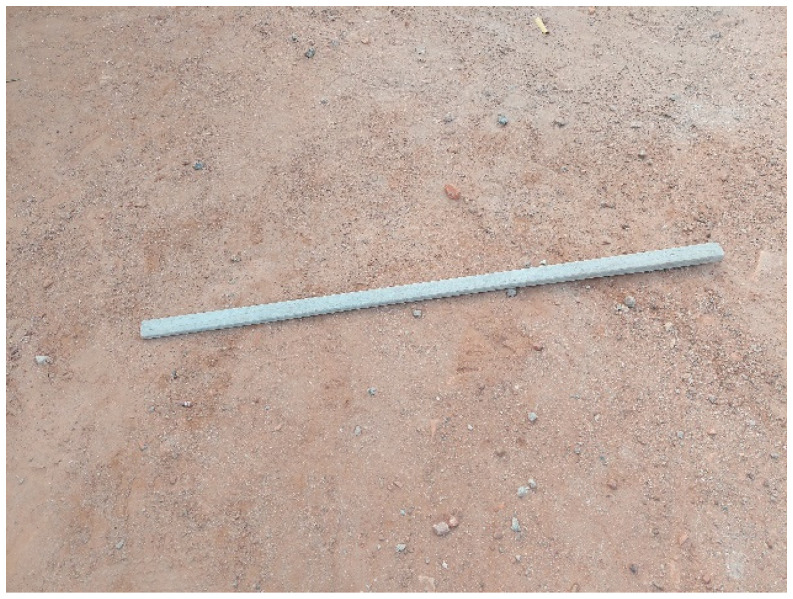
A stick lying on the floor.

Some of the adolescents with disabilities characterised their conditions as difficult, burdensome, and full of rejection after comparing the researchers’ and participants’ interpretations. One participant used a picture of a stick lying on the floor to express the daily struggles. She linked the photograph to her life and said it indicates “everyone can walk on her life”. In recounting her experience, a 19-year-old adolescent with physical impairment compared her life journey to a stick lying on the ground (Figure 1). As a stick on the ground could easily be picked up, used, and dumped at any time, so she perceives her life. This experience stemmed from the fact that several men showed interest in her but do abandon her after sexual encounter. Despite believing herself to be morally upright, she attributes her frequent rejection in relationships primarily to her disability. To make matters worse, she had previously given birth to a child with a partner who does not provide paternal support. That she finds it difficult to get any employment hence renders her vulnerable to similar exploitative advances.

**Figure 2:**
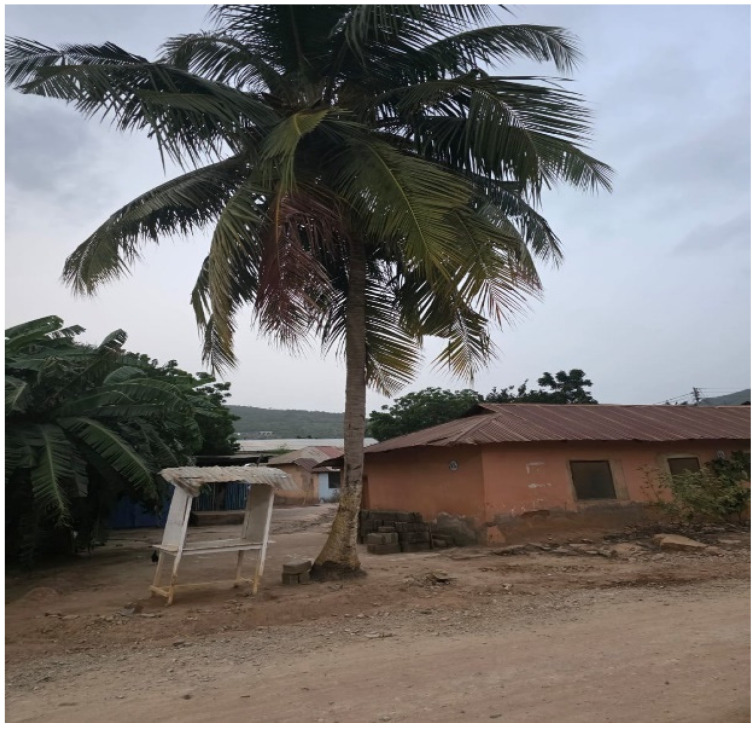
A coconut tree.

A visually impaired participant related her life to a coconut tree (Figure 2). She stated by describing the benefit people derive from the coconut tree during the rainy season and the disinterest people show to the tree in the dry season. That people come to plug the tree as it bears fruit, drink its water and eat the fruits but, after eating, they throw the husk away. Also, when the tree stops bearing fruits, people disregard its significance, except for a few people who may sit under it for shade. Even so, no one sits under it during the dry season when there are no leaves. She said “I could see with my eyes before this sickness happened to me. When I was having sight, people saw me as someone who would be a great person in the future, but now that I am blind, they believe that it is the end of my life. Some still consider me beautiful in my current state because I haven’t given birth yet, but I know if I allow myself to become pregnant, I will become a wretched thing after giving birth, which can be related to the coconut tree: once the fruit has been harvested and the leaves have been removed, it will lose its attraction”. Her adversity was born from a deep sense of rejection; after she became blind, the people who once hailed her no longer did, and that loss turned into bitterness.

**Figure 3:**
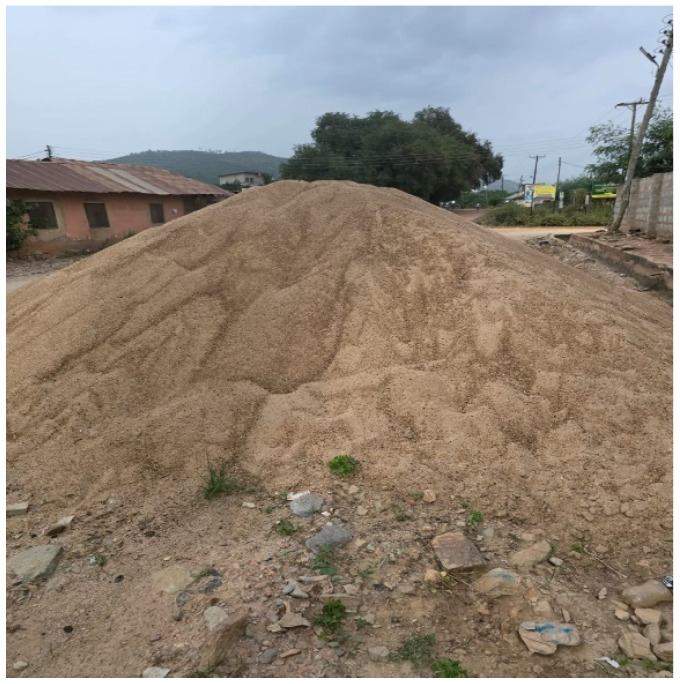
A heap of sand.

**Figure 4:**
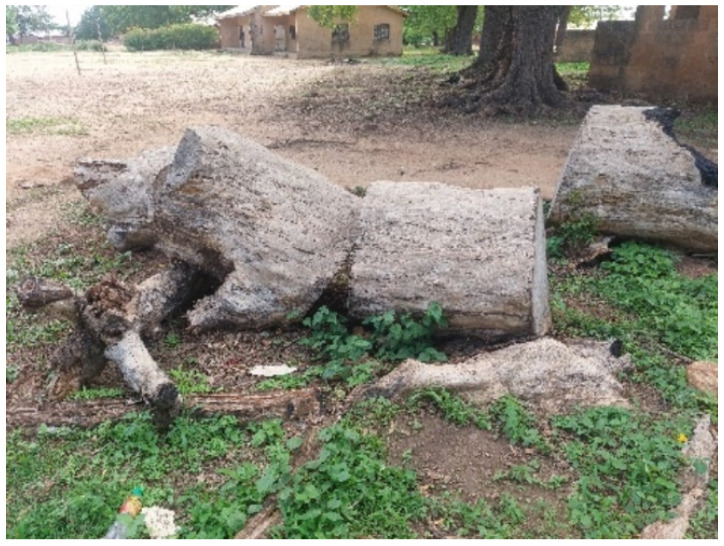
Wood logs.

**Figure 5:**
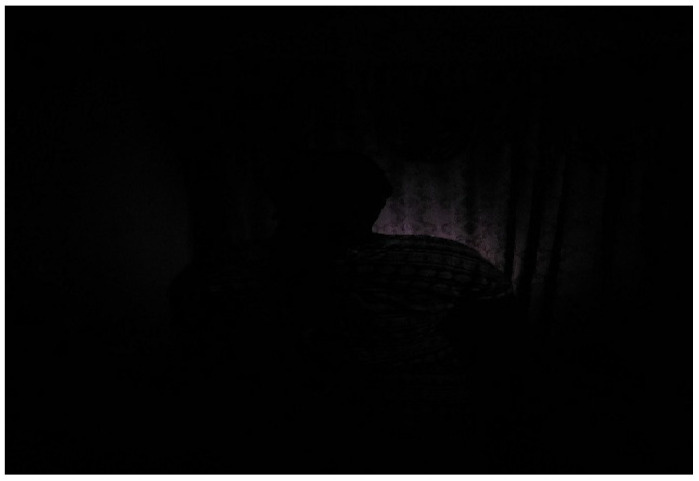
Darkness.

In another instance, a 17-year-old who is visually challenged used a heap of sand at the side of the road to explain his predicament (Figure 3). He views life as a burden because it requires him to bear more weight. Although he is fortunate to be attending school and receiving care, he feels like a burden to those who look after him, and occasionally he thinks about ending his life. However, he finds inspiration in his senior classmates who have managed to succeed in school despite having disabilities. In the same situation, during collective/group photovoices, participants used a photograph of wood logs (Figure 4) and darkness (Figure 5) to represent their lived experiences. To them, life has become a burden that they feel as though they are carrying logs of wood which are heavier and very difficult to carry. On the other hand, those who chose darkness compare their lived experiences to an unforeseen blurred future because they are physically challenged, lacking strength, and appear unfit for any productive job in the future.

**Figure 6:**
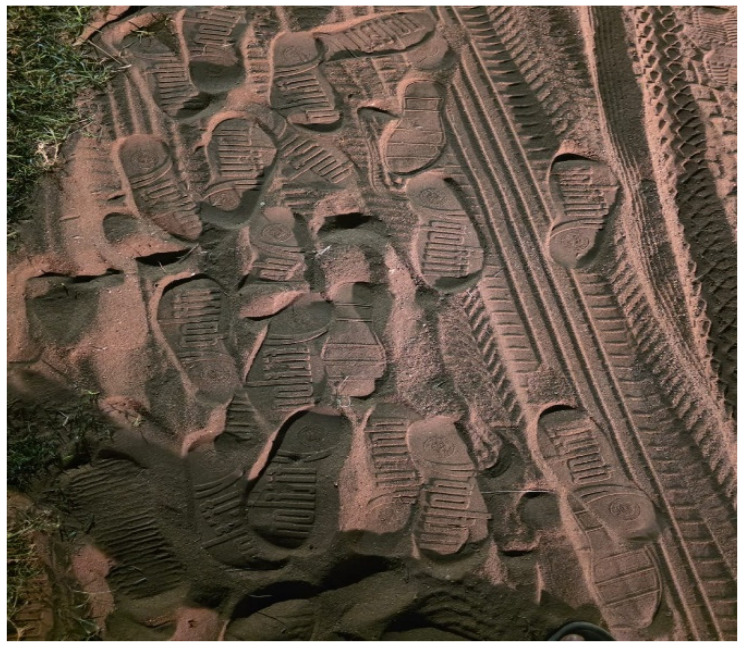
Foot path.

Consequently, a 17-year-old female participant compared her life to a foot path (Figure 6) that anyone can walk on. According to her, the disability has devalued her personality, and people make derogatory remarks about her without considering how she feels, making her feel worthless. Tracing her story, she is a physically challenged adolescent whose situation keeps on worsening as she grows up. She feels bullied by her peers and excluded or treated differently. This has made her lose her identity and status which has affected her academic and sporting activities that she used to participate in. She feels like her situation has made her powerless, and this has been affecting her mental health.

#### 3.2.2. Adolescent with Disability as Fortitude Experience

**Figure 7:**
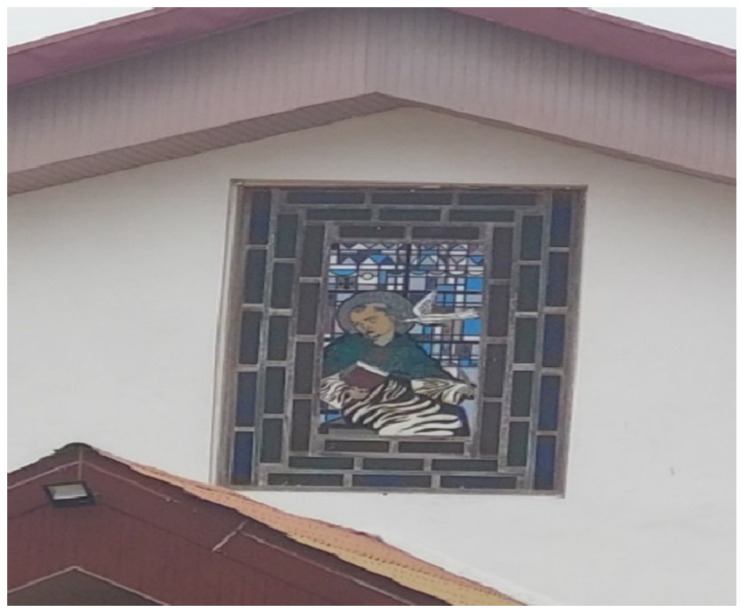
Chapel.

While some adolescents with disabilities saw their situation as challenging, there were others who were hopeful and resilience about their life experiences. One of the respondents used the photograph of a chapel (Figure 7) to explain his experience as an adolescent with a disability. The participant’s faith in God was a significant observation during the photovoice sessions. To him, the chapel represents a place where his experiences, struggles and identity are most visible and meaningful. Additionally, the church is a place of sense of belonging because he feels accepted beyond his disability. He sees his disability as a test he is battling with and it only God that can save him from that adversary. His trust in God has made him strong in health (though he feels some pains); he has always been hopeful which has raised his self-esteem and has given him more hope in life because he believes the Lord has time for everything and one day God will heal him.

#### 3.2.3. Adolescents with Disabilities as a Social Support

**Figure 8:**
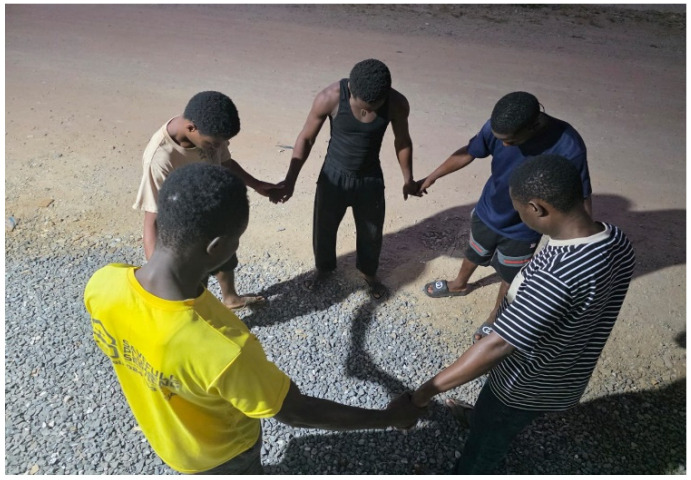
Group of people holding hands.

During one of the collective photovoice sessions, some participants said they derive their resilience from their group support network. They claim that coming together to share their experiences and singing songs keeps their hopes alive. They used one of their photographs during one of their meetings (Figure 8) to represent their experiences. To them their meetings provide a safe space to share feelings like anger, frustrations and hope for them. More so, their meetings create a sense of community in which practical coping strategies such as how to navigate through school activities and life in general were shared. Sometimes, they receive visitors who support them with empowerment programmes and provide them with assistive devices.

## 4. Discussion

This study highlights the experiences of adolescents with disabilities using photovoice methodology. The participants used different images to represent experiences in their socio-cultural environments. For example, images symbolising darkness, a stick lying on a bare floor, a coconut tree, heaps of sand, and wood logs were interpreted as representing various lived experiences. Additionally, photographs depicting a chapel and group gatherings were used to reflect coping strategies and social support, respectively.

First, we found that most of the adolescents with disabilities experienced the situation as fundamentally adversarial. Participants described their experiences as highly challenging and burdensome which is often marked by persistent feelings of rejection. This finding is consistent with studies conducted in other settings [9,25], which reported similar experiences despite using different methodological approaches. For example, studies in Ethiopia and Ghana have shown that adolescents with disabilities often have limited access to emotional support systems, experience social exclusion, face challenges in accessing healthcare, and are frequently excluded from education, particularly at the secondary school level [9,26].

Other studies have similarly indicated that adolescents with disabilities are more likely to experience bullying and violence compared to their peers without disabilities [27,28]. A notable distinction between this present study and previous research relates to participant recruitment, as this study recruited participants through schools, thereby including only adolescents enrolled in basic education. Aside from this methodological difference, the findings of prior studies are largely consistent with those of the current study. In addition, participants encountered a range of adversities that were not directly attributable to the type of disability they had. Although they lived with various impairments, including physical, visual, and hearing disabilities, their experiences were not exclusively defined or determined by these conditions.

We further observed that the experiences of participants, particularly adolescent females, were more adverse compared to those of males. This finding aligns with previous studies indicating that women and girls often face gender-based inequality, and that female adolescents with disabilities are frequently neglected, making them more susceptible to physical, sexual, and emotional abuse [29,30]. These findings revealed a significant variation that explains the different ways in which adversity is perceived and navigated. In addition, similar experiences were interpreted by another group of adolescents as opportunities for fortitude, gratitude to God and belief in Him. These divergent views highlight the heterogenous nature of experiences among adolescents with disabilities.

From a fortitude perspective, gratitude towards God emerged as a key source of strength fostered by sense of belonging within the church. Despite visibility of their struggles, some participants perceived divine provision in the form of good health, self-esteem and patience. These findings are consistent with earlier studies [31,32], which found that resilience and self-esteem were partly mediated by positive coping strategies, particularly the acceptance of events as being the will of God. Having a positive coping strategy is associated with several benefits such as the feeling of gratitude, improved health, increased self-esteem, not easily despairing, being more patient and living with less burden [33,34]. Further, we found that adolescents with disabilities articulate their gratitude towards God through meanings shaped by their religious context. In particular, the participants described the church building or space, relationships with God, church members and engagement in church activities as influential in nurturing their faith and expressions of gratitude. This finding is similar to a previous findings reporting that gratitude and faith in God are influenced by religious experience [33].

We also found that the presence of family and community support systems serves as a significant driver for bringing adolescents with disabilities together to share their experiences in order to encourage each other to live a normal life. Other studies [34,35] have found that family and community support critically influence the active participation of persons with disability in community activities.

The findings of this study should be viewed in light of some strength and limitation. The strength lies in its ability to provide insightful information which is relevant to policy and programme design. Also, the maximum variation of the sampling approach which facilitated inclusion of participants with diverse experience across a range of socio-demographic backgrounds underscores its strength. However, one has to be cautious in generalising the findings to all adolescents with disabilities in Ghana given that the participants were mainly those in school. In fact, the views expressed by the in-school adolescents in this study may not be the same as those out of school. Given that participants’ own image capture is central to empowerment in photovoice, those with visual impairments required assistance from caregivers or research assistants, thereby limiting participants’ full independent participation in the image-capturing process.

## 5. Conclusions

This study documents the experiences of adolescents with disabilities in Ghana using photovoice methodology. The findings indicate that adolescents with disabilities often encounter hostile experiences that contribute to negative self-perceptions, including feelings of hopelessness about their future. These results underscore the urgent need to establish community support systems and empowerment groups for adolescents with disabilities in Ghana as part of broader efforts to enhance their wellbeing.

## Figures and Tables

**Table 1:** Themes, samples and preliminary interpretations from the researchers.

Theme	Sample Photograph	Researcher Interpretation
Challenging	Figure 1: Photograph of a stick lying on the floor	The image suggests that there is a physical barrier, obstacle in coping with their strategies. This means that the disability blocks her path to achieving higher status in life
	Figure 5: A photograph of darkness	The image reflects inadequate support systems, lack of guidance or limited access to resources, leaving the adolescent feeling in the dark
	Figure 6: Photograph of bare ground	It means that the environment is physically present but not designed for inclusion. That is, the spaces exclude rather than support adolescents with disabilities
Resilience	Figure 3: Heap of sand	The sand means that something new will be build but right now the disability makes life harder for them
	Figure 4: Photograph of wood logs	The logs are strong and enduring. They view the logs as symbols of their own strength, persistence and ability to withstand challenges
Support Systems	Figure 2: Photograph of a coconut tree	The tree means a safety place or place of emotional safety
	Figure 7: Photograph of a chapel	The chapel represents guidance, moral support or sense of direction during a confusing stage of their life
	Figure 8: Photograph of group of people holding hands	The image represents a safe environment where communication feels easier and less judgmental

**Table 2:** Themes, samples and interpretations from the participants (adolescents with disabilities).

Theme	Sample Photograph	Participants Interpretations
Rejection	Figure 1: A stick lying on the floor	Easily picked up, used, and discarded at will by others
	Figure 2: A photograph of a coconut tree	She describes the benefit people derive from the coconut tree during the raining season and the disinterest people show to the tree in the dry season
Burden	Figure 3: Photograph of a heap of sand	Life feels like carrying the sand heap on her head every day
	Figure 4: Wood logs	He feels he has to carry these logs all by himself on a daily basis just to make ends meet
Challenging	Figure 5: Photograph of darkness	Darkness represents she cannot foresee her future because she appears unfit for any productive venture
	Figure 6: Foot path	Compared life to a foot path that anyone can walk on
Hopefulness	Figure 7: Photograph of a chapel	Our hope is in the Lord
Support system	Figure 8: Photograph of group of people holding hands	Coming together keeps us going everyday

## Data Availability

Participants consented for anonymised transcripts to be shared on request.
